# Molecular Insights into Abiotic Stresses in Mango

**DOI:** 10.3390/plants12101939

**Published:** 2023-05-09

**Authors:** Pandiyan Muthuramalingam, Subramanian Muthamil, Jayabalan Shilpha, Varadharajan Venkatramanan, Arumugam Priya, Jinwook Kim, Yunji Shin, Jen-Tsung Chen, Venkidasamy Baskar, Kyoungmi Park, Hyunsuk Shin

**Affiliations:** 1 Division of Horticultural Science, Gyeongsang National University, Jinju 52725, Republic of Korea; pandianmuthuramalingam@gmail.com (P.M.); shilphajayabalan@gmail.com (J.S.); 2 Department of GreenBio Science, Gyeongsang National University, Jinju 52725, Republic of Korea; dnrgus7165@gmail.com (J.K.); syj991008@naver.com (Y.S.); 3 Herbal Medicine Resources Research Center, Korea Institute of Oriental Medicine, Naju 58245, Republic of Korea; smuthamil66@gmail.com; 4 Department of Biotechnology, PSG College of Technology, Coimbatore 641004, India; mail4venkat1992@gmail.com; 5 Department of Biological Sciences, North Carolina State University, Raleigh, NC 27606, USA; priya6bt@gmail.com; 6 Department of Life Sciences, National University of Kaohsiung, Kaohsiung 811, Taiwan; 7 Department of Oral and Maxillofacial Surgery, Saveetha Institute of Medical and Technical Sciences (SIMATS), Saveetha Dental College and Hospitals, Saveetha University, Chennai 600077, India; baskarbt07@gmail.com; 8 Department of Horticulture Research, Gyeongsangnam-do Agricultural Research and Extension Services, Jinju 52733, Republic of Korea; parkmiya@korea.kr

**Keywords:** abiotic stress, biostimulants, *Mangifera indica*, multi-omics, abiotic stress-responsive genes

## Abstract

Mango (*Mangifera indica* L.) is one of the most economically important fruit crops across the world, mainly in the tropics and subtropics of Asia, Africa, and Central and South America. Abiotic stresses are the prominent hindrance that can adversely affect the growth, development, and significant yield loss of mango trees. Understanding the molecular physiological mechanisms underlying abiotic stress responses in mango is highly intricate. Therefore, to gain insights into the molecular basis and to alleviate the abiotic stress responses to enhance the yield in the mere future, the use of high-throughput frontier approaches should be tied along with the baseline investigations. Taking these gaps into account, this comprehensive review mainly speculates to provide detailed mechanisms and impacts on physiological and biochemical alterations in mango under abiotic stress responses. In addition, the review emphasizes the promising omics approaches in unraveling the candidate genes and transcription factors (TFs) responsible for abiotic stresses. Furthermore, this review also summarizes the role of different types of biostimulants in improving the abiotic stress responses in mango. These studies can be undertaken to recognize the roadblocks and avenues for enhancing abiotic stress tolerance in mango cultivars. Potential investigations pointed out the implementation of powerful and essential tools to uncover novel insights and approaches to integrate the existing literature and advancements to decipher the abiotic stress mechanisms in mango. Furthermore, this review serves as a notable pioneer for researchers working on mango stress physiology using integrative approaches.

## 1. Introduction

Mango (*Mangifera indica* L.) is one of the most important fruits of the tropics and subtropics across the globe. Mango is known as the ‘king of fruits’ because of its unique rich taste, nutritive value, nutraceutical bio-actives, flavor, size, and diverse color, along with aroma [1]. This tree comes under the family of Anacardiaceae. It has a small, imputed genome size of approximately 450 MB, is an allotetraploid with 2n = 40 chromosomes and is highly cross-pollinated and heterozygous [1,2,3]. Conventional crop improvement attempts have resulted in the development of three dozen new hybrid varieties, while the presence of huge genetic diversity in mango has paved the way for the identification of more than 1200 registered seedlings, hybrids, superior varieties, and rootstocks [1]. Additionally, this tree caters the food and fodder as well as fuel for a large number of populations, particularly in rural and tribal regions of developing countries. Moreover, the popular mango industry has led to the development of several processed and value-added forms of products [4]. Overall, the huge genetic diversity of mango and its importance as a food and economic resource make it a highly significant fruit tree. Field-grown plants are simultaneously confronting various abiotic stresses due to emerging environmental conditions [5]. These severe climatic factors are antagonistic to most plants, including evergreen trees, for their survival and reproduction. In addition, factors that disturb the developmental process of plants are becoming a global issue due to their negative impacts on crop productivity [6,7]. In general, plants and fruits respond to abiotic stresses by altering their molecular physiological mechanisms at gene, protein, and metabolite levels. Abiotic stress tolerance is gained by the enhancement or synthesis of antioxidant compounds and via molecular interactions at various omics levels [6].

Mango is an important tropical fruit crop affected by a range of abiotic stresses, including extreme temperatures (both heat and cold), water deficiency, salinity, and heavy metal stress. These stresses often cause morpho-physiological, anatomical, and biochemical changes, ultimately affecting the growth and productivity of mango. For instance, exposure to high temperatures and low air relative humidity can cause a reduction in the efficiency of photosynthesis, transpiration, and water potential in mango leaves [6,8]. Largely, abiotic stresses lead to the synthesis of reactive oxygen species (ROS), which can inactivate enzymes and damage vital cellular components [9]. Therefore, detailed mechanistic insight into the damaging effects of abiotic stresses is a prerequisite for developing useful horticulture tactics to counteract the negative effects. Generally, plants use various strategies, such as reduced canopy leaf area, deeper root penetration, improved osmotic adjustment, inhibition of stomatal conductance, and higher relative water content (RWC) to thwart the harmful effects of abiotic stresses [10]. In addition to these, understanding and development of new tools and methods in handling the abiotic stress response in mango are extremely inadequate, and hence its candidate genes, proteins and its functional ontology, and molecular perspectives remain understood scantly [1]. Hence, to improve crop yield, productivity, and abiotic stress avoidance, it is essential to analyze the molecular physiological cross-talks and their associated modifications by mango trees. Meanwhile, the advancement of plant science research across the world harnesses novel ways to gradually increase mango production, and global consumption is expected to increase in the future.

These issues alarm the focus on plant physiology and stress biology researchers to investigate the physiological and molecular stress dynamisms and their signal transduction network in mango. Notably, the availability of multi-omics approaches, biostimulants, and the literature information aid the mining of stress-responsive candidates and their associated functions, thereby delineating molecular insights [1,11]. The existing high-throughput approaches, the evolution of concepts, and holistic techniques utilized for molecular dissection of abiotic stress mechanisms in mango trees help in directing and manipulating the abiotic stresses and improve the overall tolerance of mango trees. These perspectives will help in unveiling the abiotic stress adaptation and tolerance, and it will allow us to obtain higher yields. Despite the importance of mango trees, to the best of our knowledge, there is no comprehensive review available on the specific aspects of abiotic stress in mango trees. Hence, we have summarized the current research to unveil advanced biological avenues aimed at overcoming abiotic stresses in mango trees. Mango abiotic stress mechanisms are discussed in relation to their molecular/genetic basis to elevate abiotic stress tolerance in diverse ways. Furthermore, stress-associated gene mining via multi-omics approaches is crucial for deciphering the tolerance mechanisms compiled in this review. In addition, biostimulants are also applied to alleviate abiotic stress tolerance. The main impact of this review is not merely to compile the baseline research on mango but rather to develop novel concepts about mango abiotic stress-resistant analyses and the possibility of making/harnessing the avenues that appear in the field of stress biology.

## 2. Abiotic Stresses in Mango

### 2.1. Drought/Water Deficit Stress

Drought is one of the most significant abiotic stress factors that negatively impact the overall growth and productivity of mangoes. The adverse physiological responses of water deficit conditions include reduced water content and water potential of the leaf, maximum turgor loss, stomatal closure, and decreased cell enlargement and growth [12,13]. Overall, water stress might affect photosynthesis, disrupt metabolism, and ultimately cause the death of plants. In mango, the presence of vegetative flushes is greatly reduced during the water deficit condition. Although drought stress for a brief period induces flowering, it shows strong inhibitory actions on vegetative growth, such as a reduction in the number of leaves in a flush, length, and leaf water potential [14,15]. Regulated deficit irrigation (RDI) has been practiced as a non-traditional method to alleviate water stress tolerance in mangos, where growth and productivity are less delicate to water deficit [16,17]. Helay et al. demonstrated the beneficial role of silicon (Si) in improving the drought stress of mango [18]. They observed that supplementation of Si in the form of K2SiO3 under drought stress alleviated the growth parameters as well as the levels of growth-promoting endogenous hormones such as IAA, GA, and CK while decreasing the level of ABA. The major mechanism of Si-mediated drought tolerance in mango cultivars was found to improve antioxidant activity. Additionally, Silva et al. used a biostimulant consisting of yeast extract and amino acids to alleviate the effects of drought stress caused by heat stress and low water availability of the mango cultivar ‘Tommy Atkins’ growing in the semi-arid region of Northeastern Brazil [19]. Recently, Shanthala et al. evaluated the effect of induced moisture stress on the rootstocks of four different Indian mango varieties and found that vegetative growth parameters, including leaf area, stem diameter, and plant height, were dramatically reduced during moisture stress [10]. However, a reduction in stomatal density and maintenance of high relative water content was recognized as the main physiological adaptations of mango rootstocks under severe water stress conditions.

### 2.2. Cold Stress

The ideal temperature range for mango is between 24 °C and 26.7 °C, and at below 10 to 12 °C, these plants begin to suffer from cold/chilling stress [20]. Mango trees are quite sensitive to cold temperatures (0–15 °C). The symptoms of chilling stress in mango fruits include pitting, rotting, lenticel darkening, irregular color development, and poor fragrance [21]. The onset of these symptoms is often interconnected with the degeneration of the cell wall membrane, lack of energy production, and increased ROS [22,23,24]. The RWC, membrane stability index (MSI), and total free amino acids (TFAA) were significantly reduced, while electrolytes leakage percentage (EC%) was increased when mango leaves were exposed to cold stress at 5–10 °C [20]. Thus, understanding the biology of low temperatures in mango could form the basis for the development of cold-tolerant cultivars and new cold-protection techniques in the future. Maturity-related chilling stress tolerance in mango has been demonstrated, wherein the chilling injury was less in yellow and pre-yellow fruit compared to green fruit [25]. The greater resilience of ripe mango towards chilling injury than unripe could be attributed to the increased antioxidant activity. In a study conducted by Sivankalyani et al. it was found that an increased accumulation of anthocyanin and flavonoids in the red peel of mangoes during the ripening stage offered greater protection against cold stress [26]. The green mangoes exhibited more chilling injuries, such as black spots and pitting, than the red mangoes. The cold tolerance mechanism exhibited by red mangoes via anthocyanin and flavonoid production was supported by another study [24]. The transcriptomic analysis of mango fruit subjected to cold stress (5 °C) showed the upregulation of candidates in the α-linolenic acid metabolic pathway leading to the oxidation of α- linolenic acid and the synthesis of methyl jasmonate (MeJA) and the oxylipin volatile compounds indicating the severity of cold stress [27]. The dynamism in the protein expression linked with chilling injuring was studied by exposing the ‘Keitt’ mangoes to quarantine hot water treatment (HWT) [28]. The study revealed that the cold stress tolerance mediated by HWT in mango was linked through the activation of heat shock proteins (HSP), enzymes of energy metabolism, synthesis of secondary metabolites such as phenylpropanoids and carotenoids, antioxidant enzymes, hormone metabolism, pathogenesis-related (PR) proteins along with enzymes of the cell wall and chloroplast metabolism.

### 2.3. Salinity Stress

In general, salinity acts as an abiotic stress factor by affecting crop plants, where the increased absorption of Na^+^ and Cl^−^ leads to ionic toxicity and osmotic stress, which results in a lack of K^+^ and Ca^2+^ and a nutritional imbalance [29]. Mango is regarded as a salt-sensitive crop [30], which results in leaf bending and burning of the tips and margins [31], and in severe situations, restricted growth, dropping of leaves, and death of tree occurs. Salinity has been shown to limit seedling growth and inhibition of chlorophyll content, reduction in CO_2_ absorption, and deficiency in nutrient uptake [32,33]. Kishor et al. proposed that paclobutrazol (PBZ) treatments boost the salinity stress avoidance/tolerance in mango by raising the photosynthetic pigments levels, K^+^ uptake, and water potential and by lowering/reducing the membrane injury index, defoliation, also the absorption and accumulation of detrimental Na^+^ and Cl^−^ ions [34]. The salt-sensitive retort varied among different mango rootstocks. For instance, the mango rootstocks such as “Olour” and “Turpentine” were reported to be saline tolerant, owing to their capacity to prevent the Cl—and Na^+^ ions uptake in tandem through greater concentrations of proline [35]. In another study, the rootstock of the genotype ‘Gomera-3’ exhibited more sensitivity towards salinity than the rootstock of the ‘13/1’ genotype. The major physiological tolerance mechanisms exhibited by the ‘13/1’ root stock include the increasing pattern of foliar K+ and proline concentration as well as the postponement of leaf abscission [36]. Recently, a study investigated the effect of sodium silicate pentahydrate and GB (Glycine betaine) in ameliorating the detrimental response of salinity stress in seedlings of a mango cultivar, ‘El-Gahrawey’ [37]. Combined application of Si and GB as the foliar spray has drastically improved the growth parameters, leaf mineral content as well as total phenolic content, thereby providing better adaptability to mango seedlings under salinity stress. Overall, these studies revealed that understanding the molecular physiological activities are essential for abiotic stress resistance in mango trees.

## 3. Abiotic Stress-Induced Physiological Activities in Mango

The prevalent abiotic stressors that challenge the typical growth and development of mango plants include low or high temperatures, salt, drought, heavy metals, etc. [38]. Among these, temperature fluctuation has a major impact on the frequency, extent of flowering, development of fruit, and quality of mango. On the other hand, water stress has some benefits on mango, specifically during the flowering stage. Water stress induces and accelerates the flowering process. Salt stress in mango can impair cultivation as the discrepancy in ion exchange can cause an imbalance in the nutrients, a reduction in the growth and fruit-bearing capacity, and an overall decline in the yield [39]. Recent investigations have revealed that abiotic stress tolerance in mango is conferred by eukaryotic initiation factors (eIF) [40].

Abiotic stress conditions induced by adverse environmental conditions ensure an instant change in the physiology of fruits [41]. Often, in mango, the fruits seem wholesome and intact in their external appearance, but only the mesocarp of the fruit will be spoilt, which is generally known as spongy tissue disorder [42]. Mesocarp ripening is crucial for the healthy external appearance of the fruit as well as its taste and flavor. It was detected that numerous transcriptional and metabolic alterations happen in the course of this spongy tissue disorder [43]. Oak et al. comprehended the comparative transcriptional modifications in Alphonso mango fruit under normal and diseased conditions through RNA sequencing, proteomics, and real-time PCR techniques. Various transcription factors (TFs) related to stress are found to be co-expressed with the transcripts associated with flavor and ripening properties [44]. With the multi-omics approach, it has been highlighted that the mechanisms involved in cell wall synthesis, ethanol and flavonoid biosynthesis, flavor formation, and fruit ripening are altered in the spongy tissue disorder that impedes the quality and taste of the Alphonso fruits [44].

In another study conducted by Jardine et al. the high temperature and light stress induced the emission of isoprene oxidation products into the environment. This study suggests that the presence of abiotic stress not only impacts the physiology of the plant but also contributes to the contamination of the environment [45]. Heat waves are another predominant abiotic stressor that inhibits the growth, development, and quality yield of mango. It has been shown that during exposure to heat waves, the mango fruit pulp extracts deteriorated in their radical scavenging activity. Expression of genes involved in oxidative stress, senescence, circadian rhythm, glycolysis, biosynthesis of secondary metabolites, flavonoids, monoterpenoids, etc., was found to be altered [6].

One of the common post-harvest abiotic stresses is the treatment of fruits to enhance their storage and preservation. However, it has been reported that such treatments can interfere with the quality and taste of the fruits after storage. Luria et al. demonstrated that hot water brushing of mango fruits showed variations in the gene expression related to fruit quality and disease resistance [46]. By analyzing the transcriptome of untreated and treated fruit peels, alterations in the three main categories of genes were unraveled, viz., genes involved in abiotic stress responses, photosynthesis and degradation of chlorophyll, and genes associated with sugar and flavanoid metabolism. This finding demonstrates that several biochemical and physiological consequences can occur post-harvest, and those can impact the quality and originality of the fruits [46]. In addition to the above, cold storage can also significantly impact the physiology and quality of the mango fruit. As mango is a tropical fruit, it is highly susceptible to cold temperatures. When stored under a temperature below 12 °C, mango fruits can undergo several alterations in their physiological and metabolic pathways, which can lead to various cellular dysfunctions. Some of the variations include elevation in ethanol production and respiratory rate, alteration in cell structure and membrane dysfunction, enzyme inactivation, and ROS production. Transcriptome analysis of mango fruit under chilling stress revealed the stimulation of defense response signaling, lipid peroxidation, and elevation of phenylpropanoids biosynthetic pathway [26].

Researchers around the world are exploring various strategies to alleviate the abiotic stress conditions in mango plants grown in greenhouse and natural field environments [19,47,48]. These approaches include the use of nanomaterials [47], accumulation of amino acids such as proline, alone or in combination with algal extract [48] or yeast extract and other micronutrients [19], Si [18], potassium silicate, and tocopherol [49], among others. With the aid of multi-omics approaches and bioinformatic analyses, researchers are endeavoring to uncover the physiological and biochemical changes that occur under various abiotic stresses. This knowledge helps in identifying the molecular cross-talks and abiotic stress-responsible genes, which can be used to develop methods for overcoming abnormal growth, development, and yield due to abiotic stress in mango trees [40,44,50,51].

## 4. Role of Abiotic Stress-Responsible Genes

Plant responses to various abiotic stress are highly interconnected and complex due to the association of clusters of candidate genes and TFs. TFs are the pivotal players which can interact with many transcriptional regulators and are involved in plant stress dynamisms and cascading diverse signal transduction pathways [51,52]. They are also involved in transcriptional reprogramming, altering biomolecules, differentiation, and developmental processes in plants [53]. In addition, plant TFs consist of special structures associated with *cis*-regulatory elements of stress-responsive players and can differentially regulate the dynamism of many downstream genes to stimulate and enhance the plant stress responses, including both biotic and abiotic stresses [53]. To date, 58 TF families have been categorized approximately [54]. Of these, MYB, NAC, WRKY, HSF, DREB, and AP2-EREBP are the notable TFs families. The family members of these TFs stimulate physiological and cellular processes, including callus formation, differentiation, protein storage in seeds, secondary metabolites biosynthesis, directing the plant cell metabolisms, plant development, and enhancing tolerance mechanisms against abiotic and biotic stresses [52].

In addition, defensive mechanisms play significant regulations in improving the quality of mango fruit during various abiotic and biotic stress responses. Notably, abiotic stresses alter the genetic and metabolic cross-talks, cause cell death, increase lipid peroxidation and sugar metabolisms, inhibition or decrease the photosynthesis efficacy, and cause salt accumulation in mango. It affects plant growth, imbalance of nutrients, inhibition of nutrients uptake from the soil, leaf injury, and reduction in the leaf area and height, followed by a reduction in yield and overall quality of plants [27,55]. These physiological responses are associated with and controlled by genes as they regulate various biomolecules, biosynthesis of proteins, and abiotic stress responses. A few essential genes in response to abiotic stresses, including cold stress and their molecular functions in mango, were given in Table 1. In general, plant researchers face a challenging task in understanding the intricate environmental responses of plants to various abiotic stresses. Developing abiotic stress-resistant mango plants poses an additional challenge. To address this, multi-omics approaches combined with computational biology uncover the abiotic stress responsible players, and it can reveal the complexities of abiotic stressors, leading to the identification of new avenues for mango research.

## 5. Omics Approaches to Dissect the Abiotic Stress-Resistant Mechanisms in Mango

**Figure 1:**
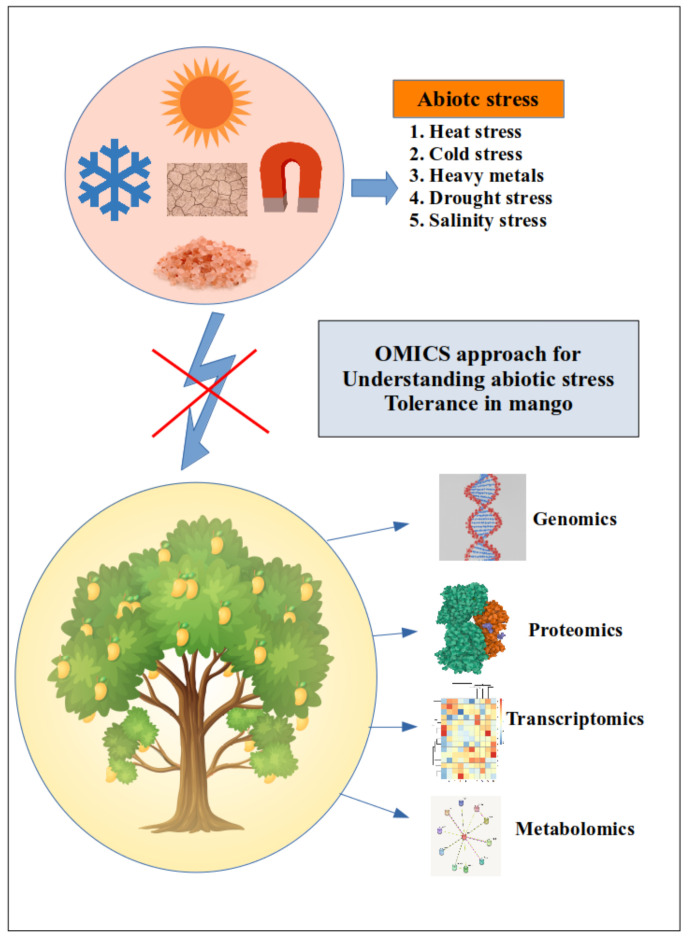
‘OMICS’ methods for understanding the mechanism of abiotic stress tolerance in mango.

In order to decipher the mechanism of resistance to abiotic stress in mango, recent studies used multi-omics approaches (Figure 1) such as genomics, transcriptomics, proteomics, and metabolomics.

### 5.1. Using Genomics to Investigate Abiotic Stress Tolerance in Mango

Genomic techniques in mango are useful for accelerating trait-specific varietal development via precision breeding efforts [61]. The WD40 protein family is one of the amplest protein families in all higher plants. They play critical roles in plant growth and regulation. Mango contains a total of 315 WD40 protein members (*M. indica* L.). Researchers discovered a novel protein called TRANSPARENT TESTA GLABRA 1 using the Bimolecular fluorescence complementation (BiFC) assay (*MiTTG1*). The MiTTG1 protein also interacts with other proteins in mango, including *MiMYB0*, *MiTT8*, and *MibHLH1*, resulting in the creation of a new ternary regulatory complex (MYB-bHLH-WD40). Furthermore, the transgenic lines of *MiTTG1* were found to be better adjusted to abiotic stresses, including mannitol, salt, and drought stress, by modulating root hair development [62].

On analyzing the expression profile of genes responsible for abiotic stress tolerance during the growing phase of mango fruit, it was observed that several genes (*MiGAD*, *MiNRX1*, *MiGI*, *MiGSTF6*, *MiWun1*, *MiCAT1*, and *MiPER42*) which regulate abiotic stress dynamisms exhibited a significant up-regulation. When prone to stress of high light, there had been an initiation of anthocyanin biosynthesis which confirms tolerance to the plant by alleviating the ROS that correlated with the up-regulated pathways of fructose and mannose degradation, malate-aspartate shuttle, and galactose metabolism [6]. SQUAMOSA promoter binding protein-like (SPL) genes are known to play important roles in plant growth and developmental processes. In the mango genome, a total of 26 SPL family members were identified and analyzed. These genes were expressed in response to exogenous gibberellin-3 (GA_3_) and prohexadione-calcium (Pro-Ca) treatments. In addition, the *MiSPL13* gene was found to be up-regulated in flowers and highly expressed in buds during GA_3_ and Pro-Ca treatments. The full-length cDNA sequence of MiSPL13 was 1116 bp, encoding 372 amino acids. Overexpression of *MiSPL13* increased the expression levels of other genes, such as the *AtAP1*, *AtSOC1*, and *AtFUL*, and thereby significantly enhanced the tolerance of plants against various abiotic stresses, including drought, abscisic acid (ABA), and GA_3_ [63].

*Mother of FT* and *TFL1* (*MFT*) is a part of the phosphatidylethanolamine-binding protein (PEBP) family, which plays prominent roles in seed development, response to stress, and flowering time regulation. According to Lu et al. [64], the MFT homologous gene *MiMFT* was discovered in the ‘SiJiMi’ mango cultivar. The phytohormone, plant growth, plant development, and abiotic stress-responsive elements were all present in the *MiMFT* promoter. *MiMFT* was found in significant amounts in the seeds and responded strongly to polyethylene glycol (PEG) and NaCl treatments. Furthermore, salt and drought tolerance increased significantly with transgenic lines overexpressed *MiMFT*, and these plants showed decreased responsiveness to ABA, with massively higher expression of stress-related and ABA signaling pathway genes. Apart from these genes, nine other genes with known functions involved in numerous abiotic stresses tolerance in mango were confirmed by Luo et al. using quantitative reverse transcription polymerase chain reaction (qRT-PCR) in leaves and stems under cold (4 °C), salinity (NaCl), polyethylene glycol (PEG, MW 6000), and heavy metal treatments in different time intervals [60].

### 5.2. Transcriptomics Approach for Studying Abiotic Stress Resistance in Mango

The study of gene expression patterns and RNA levels in biological samples, known as transcriptomics, has significantly improved our understanding of the molecular mechanisms behind diverse biological processes in mango, including fruit growth, ripening, and response to biotic and abiotic stress [44,65,66]. Among these processes, the transcriptome level response of mango to several abiotic stimuli has been well studied. The transcript levels of many C2H2-type zinc finger proteins are enhanced in response to diverse abiotic stress factors, including cold, salinity, drought, osmotic stress, and oxidative stress, according to transcription profiling of mango [60]. Apart from C2H2-type zinc finger proteins, studies have found numerous genes that are differently expressed in stress response pathways, including those involved in ABA signaling, osmotic control, and antioxidant defense. Additionally, many potential genes, such as those encoding aquaporins (AQPs), which are involved in water transport, may play essential roles in controlling the drought response in mango [67].

In recent years, several studies have investigated the role of potassium transporters in drought stress response in mango trees. For instance, a study by Tan et al. investigated the expression of potassium transporters in the leaves of drought-tolerant cultivar of mango under drought stress. They found that the expression of genes encoding potassium transporters, particularly those involved in the uptake and translocation of potassium such as *MiHAK5.2*, *MiHAK1.1*, *MiTPK2.1*, *MiKAT1.1*, and *MiAKT6*, was significantly up-regulated [68]. In addition, genes that regulate flowering in mangoes are implicated in drought tolerance. A study by Liu et al., successfully demonstrated the upregulation of homologs of *CONSTANS* (a flowering regulator), *MiCOL16A*, and *MiCOL16B*, in response to drought stress in mango [69].

Next to drought, salinity is a major abiotic stress factor that can affect the growth of the mango plant. While mango is generally sensitive to salinity, transcriptomics studies have shed deep insights into the transcriptome-level regulations involved in salt-tolerant cultivars of mango. Gene expression studies on the leaf samples of the siji cultivar of salt-tolerant mango showed the upregulation of several eukaryotic translation initiation factors (eIFs). The eIFs play a very important role as regulators of protein synthesis, and they are strongly associated with salt tolerance in several plants. Among the 18 eIF genes associated with salt tolerance, *MieIF1A-α*, *MieIF3sB*, and *MieIF5* are the most up-regulated genes in the siji cultivar [40]. These genes also seem to play a vital role in responding to low temperatures and osmotic stress [70]. The increasing interest in the cultivation of mango in low-temperature regions has led to the transcriptome profiling of several low-temperature-tolerant mango varieties, including the chilling-resistant Kiett cultivar, which is widely grown in the Jinsha river valley, China. The results of transcriptome profiling indicate the presence of 1123 differentially expressed genes (DEGs), with *PLD1* and *WRKY70* being significantly up-regulated after 9 h of exposure to a chilling condition (4 ºC) [71]. Apart from these DEGs, TFs such as *MYB73*, *NCED2*, and *HLH162* were found to be up-regulated in mango leaves during exposure to low temperatures. These findings suggest the existence of a complex multi-gene expression regulation system in mango trees to resist abiotic stress.

### 5.3. Proteomics Approach for Revealing Abiotic Stress Tolerance in Mango

The basis of differential-expression proteomics is comparing the composition of different proteomes. The most common situation in the research of plant abiotic stress is the comparison of proteomes isolated from plants that are not stressed (referred to as control plants) with the equivalent proteomes when certain plants are stressed. Other situations include comparing the proteomes of two separate genotypes or plant species with different degrees of tolerance to a specific stress agent [72]. A significant amount of data on the complete, comparative, and differential transcriptome and proteome of mango tissues, such as pulp, peel, and leaf, has been generated by a few studies [11]. A new, environmentally friendly substance known as -aminobutyric acid (BABA) prepares plants’ immune systems to withstand various stresses. However, the molecular processes underlying BABA-induced priming defense remain unknown. Li et al. investigated the priming mechanism of BABA-induced resistance using an iTRAQ-based proteomics method which is based on an interaction system among mango and *Colletotrichum gloeosporioides*. BABA treatments effectively decelerated the spread of *C. gleosporioides*-induced anthracnose in mango fruit, according to the findings [73]. The increased pathogen response in BABA-primed mango fruit after *C. gleosporioides* inoculum may be explained by various protein accumulations involved in secondary metabolism, defense signaling, and response, regulation of transcription and transcriptional, and post-translational modification of proteins.

The proteomics of tolerance to chilling injury in mango peel was studied by Salazar-Salas et al. [28]. The study discovered that 26 proteins are highly expressed in the mango after quarantine hot water treatment. Heat shock proteins, enzymes involved in energy metabolism, antioxidant enzymes, and PR proteins were all found to be highly expressed during stress.

Proteins with WD40 repeat domains appear to be required for a wide range of biological activities, including cytokinesis, cell expansion, cell division, meristem employer, and bone formation. As a result, the N- and C-termini of WD40 proteins contain a tryptophan–aspartic acid pair and a glycine–histidine pair, which are defined as the central residues from several important motifs (40–60 amino acids) [74]. The seven-bladed propeller domain repeats of the WD40 protein motif act as a scaffold for a variety of protein–protein interactions and promote the formation of efficient complexes. In Arabidopsis, a different WD40 protein called XIW1 connects the stable ABI5 and ABA responses. Wheat abiotic stress responses are highly correlated with *TaWD40D*. It is crucial to explore in further detail the qualities of WD40, their significance, and how they can be of potential use for mangoes [62].

### 5.4. Role of Metabolomics in Understanding the Abiotic Stress Tolerance in Mango

Mangoes can develop heat stress tolerance through genomic and metabolomic interactions, the production of antioxidant molecules, or an increase in these molecules. Plants and fruits typically adjust their antioxidant status at the metabolomic level when subjected to heat stress [60]. Biological interactions cause metabolic responses in the fruit. Cell metabolites reveal a cell’s complete physiological parameters [60]. Presumed metabolites are representatives of numerous primary and secondary metabolic pathways that may play a role in fruit preservation. The fruit’s physiology worsens due to a decrease in antioxidants and enzymes that nullify ROS in the mesocarp. ROS are produced as a result of metabolic aberrations in the tricarboxylic acid cycle (TCA) and gamma amino butyric acid shunt, which stress the mesocarp [75].

The interactions of various biomolecules, such as lipids, proteins, carbohydrates, and nucleic acids, play a crucial role in various biological processes of living organisms, including mango trees. During typical mango ripening, amylase digests mango starch into less complex carbohydrates such as glucose, fructose, and sucrose in the pulp. Heat reduces the activity of these enzymes, which affects the fine-tuning of sugar metabolism and, as a result, slows glucose metabolism in the spongy tissue. There were fewer intermediate metabolites from phenylpropanoid or shikimate pathways in the spongy tissue. As the fruit ripened, the concentration of phenolic compounds such as ferulic acid, chlorogenic acid, naringenin, and coniferin in the spongy tissue increased. Scanning electron microscopy revealed thick parenchyma cells and starch accumulation inside the mature raw stage of the mango’s healthy mesocarp, as well as starch granules and parenchyma cell wall thinning inside the ripe tissue. The concentrations of magnesium, zinc, and iron are higher, while calcium, sodium, and potassium are lower. The mango fruit is stressed, as evidenced by higher amino acid concentrations, lower protein content, and lower enzyme profiles discovered in the spongy tissue mesocarp [76].

Abiotic factors were discovered to affect the metabolic reactions of compounds such as sugar alcohols, mono- and disaccharides, and amino acids, particularly proline, polyamines, and TCA participants. The chemosensory phenotype and flavoring profile of food products have been represented by the analysis of the aroma and taste-active substances that are controlled by the genes, the expression of which is altered or even produced by biotic or abiotic stress challenges [77]. On the whole, the omics approaches are the essential platforms for analyzing deeper molecular insights and also uncover novel avenues.

## 6. Role and Types of Biostimulants in the Alleviation of Abiotic Stress

### 6.1. Biostimulants

**Figure 2:**
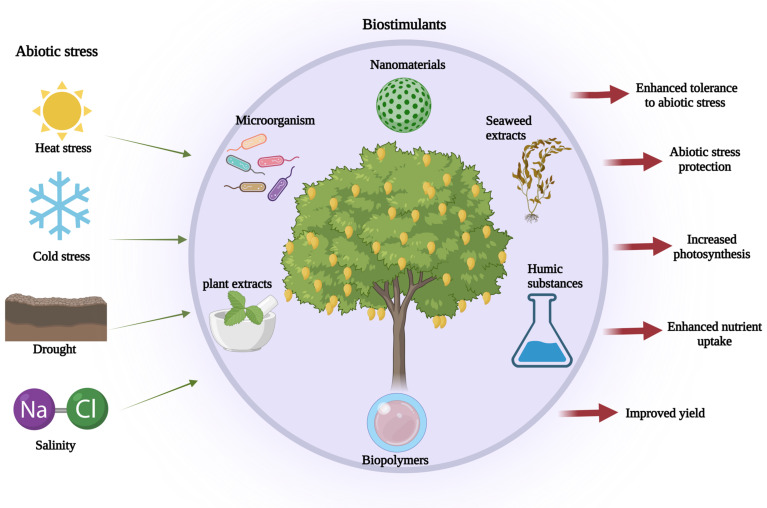
Role of biostimulants in abiotic stress enhancement with improved quality and yield of mango trees (Image created in BioRender.com accessed on 18 March 2023).

Biostimulants easily replace traditional stimulants such as pesticides and biofertilizers due to their distinctive role in controlling growth and development [78]. They have been found to improve crop yield and quality more effectively than traditional stimulants and are defined as substances or microorganisms that stimulate nutrient uptake and abiotic stress tolerance and enhance crop quality and yield (Figure 2). Biostimulants, both microbial and non-microbial, trigger various molecular, physiological, anatomical, and biochemical responses in plants, leading to increased tolerance against abiotic stress [79,80,81,82]. These stimulants can be a single or a mixture of substances derived from natural resources or microorganisms that augment the crops’ growth and development. Typically, proteins, enzymes, amino acids, and micronutrients are used as biostimulants, as well as natural stimulants such as phenols, salicylic acid, humic and fulvic acids, or protein hydrolases [83]. Comparatively, biostimulants are differ from fertilizers by assisting plants in the acquisition of nutrients. For instance, Colla and Rouphael proposed three microbial types of plant biostimulants [84], such as plant growth-promoting rhizobacteria [85], arbuscular mycorrhizal fungi (AMF) [86] and *Trichoderma* spp. [87]; six non-microbial types of biostimulants include (i) humic and fulvic acids [88], (ii) chitosan [89], (iii) protein hydrolysates [90], (iv) seaweed extracts [91], (v) phosphites [92], and (vi) Si [93].

### 6.2. Biostimulants and Abiotic Stresses in Plants

To manage the different abiotic stresses, plants can simultaneously activate several signaling cascades, resulting in cellular and molecular physiological dynamisms [94,95]. Recently, global research groups have reported the application of biostimulants to alleviate plant growth and development and enhance diverse abiotic stress tolerance. In growing mango trees, shoot maturation is an important phase that stimulates various morpho-physiological responses, including the induction of floral buds during abiotic stress conditions. Under unfavorable climatic conditions, the application of biostimulants may help to recover from shoot maturation. In particular, *Ascophyllum nodosum* algae extract promotes beneficial effects on plant physiology, although the more considerable effects are associated with improving tolerance to abiotic stresses [96].

### 6.3. Types of Biostimulants

In recent years, several research groups have reported various types of biostimulants based on their origin, component, or mode of action [81,97]. It can be classified into five types based on raw materials such as seaweed and plant extracts, humic substances, microorganisms, nitrogen-containing compounds, and inorganic compounds [98].

#### 6.3.1. Seaweed Extracts

Seaweeds are macroscopic, multicellular marine algae that belong to the brown, red, and green algae taxonomic groups. In ancient times, biostimulants were treated as natural fertilizers in agriculture fields [91]. Seaweed extracts (SWEs) are now considered essential formulations as plant growth-promoting agents to develop tolerance to salinity, drought, and heat. The majority of commercial products are derived from brown algae (*A. nodosum*, *Durvillaea potatorum*) and red algae (*Lithothamnium calcareum*) [99]. SWEs target many signaling pathways to improve tolerance under stress conditions; however, the exact mechanism behind these effects is still poorly understood. Recently, SWEs have been used to enhance resistance to cold/chilling stress. Among the tested SWEs, only extracts with zinc (Zn) and manganese (Mn) were proficient in enhancing cold tolerance through improving ROS responses [100]. Cold tolerance is improved by the extracts of *A. nodosum* [101]. Algal extracts and SWE-based cytokinins are used to improve the salinity and heat stresses on Kentucky bluegrass (*Poa pratensis* L. cv. Plush) and creeping bentgrass (*Agrostis stolonifera* L.), respectively [102]. Mohamed and El-Sehrawy, state that the foliar application of SWEs alleviates fruit yield, size, and quality [103]. Similarly, *A. nodosum* extracts are used to augment shoot maturation in mango cv. Palmer has grown in the semi-arid region as an alternate agent to commercial K (potassium) fertilizer paclobutrazol [96]. Furthermore, biostimulants containing L-α and free amino acids, soluble nutrients, and *Lithothamnium* sp. algae extract benefit the nutritional value and increase the fruit production of mango ‘Kent’ [41].

#### 6.3.2. Plant Extracts

Biostimulants that are derived from plant extracts that are rich in secondary metabolites aid in activating the physiological responses of plants [104]. For example, extracts from borage (*Borago officinalis*) have been shown to improve plant metabolism by increasing photosynthetic activity, leaf pigments, and fresh weight of the lettuce plants (*Lactuca sativa*) [105]. One possible mechanism for the improvement of crop production is attributed to the development of mineral nutrient availability and uptake. In mango cv. Fagri Kalan, foliar spraying of two plants extracts (10% roselle and 5% garlic) along with algae extract (2%) improved many fruiting measurements such as fruit set, yield, retention and quality, growth, and leaf nutritional status [106].

#### 6.3.3. Humic Substances

Humic substances (HS), which include fulvic acids, humic acids, and humins, are natural constituents of organic matter in the soil and are also produced by the decomposition of plants, animals, and microorganisms [83]. HS can induce the anatomical structure development of plant roots, and their biostimulant effects are responsible for enhancing nutrient absorption, water intake, and tolerance to environmental stress. Generally, the biostimulant effects of HS refer to the enhancement of micro and macro-nutrient uptake by roots through diverse physiological mechanisms. Owing to the increased cation switch ability of the soil containing the polyanionic HS and the improved availability of phosphorus by HS impeding with the precipitation of calcium phosphate [97,98,107]. Moreover, humic acid at a concentration of 7.5 mL/L has been found to increase growth parameters of Alphonso mango nursery grafts such as plant height, plant spread, leaf area, root length, girth at collar, dry matter production, and a number of secondary, and tertiary roots [108].

#### 6.3.4. Chitosan and Other Biopolymers

Biopolymers synthesized by living organisms can be used as active organic compounds against abiotic stresses. Among these, chitosan, the second most abundant polymer after cellulose, has garnered considerable interest due to its eco-friendly and inexpensiveness. Chitosan has frequently been employed as a biopolymer to alleviate stress tolerance mechanisms, including both biotic and abiotic stresses. For instance, in maize plants, chitosan has been found to induce a level of tolerance to water stress, as well as improved photosynthesis and antioxidant systems activity [109,110]. Similarly, wheat seedlings coated with chitosan exhibited improved drought tolerance levels by altering the molecular physiological mechanisms, leading to better seed germination, yield, plant growth, and root expansion [111]. Additionally, foliar spraying of nano-chitosan (5 mL/L) on mango trees enhanced the growth and fruit quality and also showed more resistance to malformation [112].

#### 6.3.5. Microorganisms

Microorganisms isolated from soil, plants, and other substances have the potential to increase plant growth and crop productivity through direct and indirect physiological and metabolic activities. Plant growth-promoting microorganisms (PGPMs) have beneficial effects on fruit quality by enhancing the level of proteins, polyphenols, sugar content, antioxidant properties, and anthocyanin pigment production. This can be helpful for farmers to enhance agricultural production [113]. Microbes can augment nutrient uptake via nitrogen fixation, nutrient solubilization, and alterations in hormone levels by stimulating plant hormone biosynthesis. Moreover, microbes can increase tolerance to abiotic stresses and produce volatile organic compounds (VOCs) that directly affect plants. Plant growth-promoting rhizobacteria (PGPR) play a pivotal role in ameliorating plant signaling dynamics to abiotic stresses by inducing molecular physiological activities [85,114].

#### 6.3.6. Inorganic Compounds and Nanomaterials

Inorganic elements such as Si, selenium (Se), aluminum (Al), cobalt (Co), and sodium (Na) can promote plant growth [83]. Si provides mechanical support to the tissues and increases fruit firmness. Additionally, Si is a physical barrier to prevent fungal and insect attacks [115,116]. In addition to inorganic elements, nanoparticles, and nanomaterials can act as potential biostimulants. The structure and nature of the nanomaterials determine the biostimulant properties, and the interaction between plants and nanoparticles can change the transport of ions and metabolites and also regulates plant metabolisms [117]. Moreover, nanoparticles and nanomaterials release iron or carbon that might be useful for plant metabolism. For example, Elsheery et al. confirmed the efficacy of nanoparticles on plant morphology and biochemistry [47]. Zinc oxide (ZnO) and silicone nanoparticles (nSi) exhibited a promising effect on mango trees (cultivar Ewais) under salinity stress conditions. The combined application of 100 mg/L ZnO and 150 mg/L nSi enhanced the uptake of nutrients and carbon assimilation. Consequently, the plant’s defense mechanisms were enhanced, and overall plant productivity and fruit quality were positively transformed under stress conditions. Overall, the exogenous application of biostimulants enhances plant differentiation, growth, and stress tolerance in mango trees.

## 7. Conclusions and Perspectives

Abiotic stresses are predominant factors that negatively impact overall plant growth, resulting in yield loss in mango trees around the globe. Despite its importance, detailed reports on the molecular crosstalk involved in abiotic stresses in mango are still lacking. Hence, to improve mango growth and yield during abiotic stresses, novel platforms, and methods are essential to unveil the molecular physiological machinery, develop new cultivars through breeding programs, and decipher these bottlenecks. This review outlines the main discoveries and research on key players, TFs, stress physiology, high-throughput omics, and biostimulants in mango tree systems under diverse abiotic stress conditions. These approaches and research knowledge have allowed us to delineate the novel molecular mechanisms of abiotic stresses interconnected with candidate genes and their role and functional regulations in mango plants. In addition to these advanced techniques, more attention should be paid to the molecular connections between the signaling mechanism involved in abiotic stress responses. It will aid in unveiling more molecular insights related to interactions among the abiotic stress-responsive genes and their associated abiotic stress-signaling pathways. Furthermore, integrating these approaches with genome-based breeding technologies and CRISPR/Cas9-based genome editing is the significant groundwork for ameliorating abiotic stress tolerance and enhancing yield in mangoes. Overall, this groundwork will also enable future directions by torch-bearing the questions related to the role of current investigations in abiotic stress tolerance in mango.

## Figures and Tables

**Table 1:** The details of various stress-responsive genes and their molecular mechanisms of abiotic stress tolerance in *M. indica* L.

Abiotic Stress	Genotype Used	Abiotic Stress-Responsive Genes	Type of Study Involved	Plant Tissue/Stage Showing Upregulation	Role/Function of Genes	References
K^+^ depletion, salinity, and PEG treatment	Guire 82	*MiHAK* genes (1–18) *MiHAK14* exhibit tolerance to K^+^ depletion, salinity	Isolation and characterization and expression profile analysis of *MiHAKs,* overexpression of *MiHAK14* in *A. thaliana*	Upregulation of various *MiHAKs* in root tissues under abiotic stress	*MiHAK* genes belong to KT/HAK/KUP family that encodes K^+^ transporters which provide resistance to salinity, drought, and heavy metal stress	[51]
Salinity and drought	Jin Huang	*MiCOL* genes	Genome-wide identification of *CO* genes, expression pattern analysis of *MiCOL* genes, and overexpression of *MiCOL9* genes in *A. thaliana*	Higher expression of *MiCOL9A* and *MiCOL9B* genes in leaves after 12 h of abiotic stress treatment	*COL* candidate gene in the photoperiod pathway played a role in the regulation of flowering and abiotic stress response	[56]
Low temperature, drought, and salinity	Siji	Family members of *Mi14-3-3* gene (*Mi14-3-3-A1*, -*A2*, *-B1*, *-B2*, *-C1*, *-C2*, *-D1*, *-D2*, *-E1*, *-E2*, *-I1*, *-I2*, *-6A*, *-6B*, *-7A*, *-7B*)	Genome identification and gene expression profiling using qRT-PCR	*Mi14-3-3-A1-* young stems *Mi14-3-3-6A*, *Mi14-3-3-C1* and *Mi14-3-3-D1*—adult leaves *Mi14-3-3-E1*—flowers *Mi14-3-3-I2*—buds *Mi14-3-3-A2*, *Mi14-3-3-D2* and *Mi14-3-3-7B*—young leaves	Opening of stomata, root movement, plant growth and development, hormone signaling, morpho-physiological metabolisms, and stress responses	[57]
Heat	Chaunsa White	*MiGAD*, *MiNRX1*, *MiGI*, *MiGSTF6MiWun1*, *MiCAT1* and *MiPER42*	RNA-Seq analysis, gene expression analysis using qRT-PCR	Mango fruits after 79 days of flowering (79DAF)	The enzymatic and non-enzymatic antioxidant activity involved in ROS homeostasis and circadian rhythm control	[6]
Cold, osmotic, and salinity	Siji	*MieIF* genes (particularly *MieIF1A-α*, *MieIF3sB*, and *MieIF5* were more strongly expressed during salinity, cold and osmotic stress, respectively)	Transcriptome analysis, functional analysis by overexpression of *MieIF1A-α* in *A. thaliana*	Leaves of one-year-old seedlings at various time points (0, 6, 12, 24, 48, 72 h)	Protein synthesis, translation initiation, virus resistance, vegetative and reproductive growth, and stress responses	[40]
Cold, drought, and salinity	Siji	*MiRab5*	Isolation, characterization, and gene expression analysis of *MiRab5*	Higher expression in younger leaves and stems as well as in later stages of fruit ripening	Regulate the fusion of vesicles with target membranes via conformational changes, fruit ripening, and stress responses	[58]
Cold, drought, and salinity	Siji	*MiASR*	Molecular cloning, characterization, and qRT-PCR analysis of *MiASR* gene	leaves and stems at various time points (0, 24, 48, and 72 h)	Plant development, fruit ripening, post-harvest storage, biotic and abiotic stress responses	[59]
Cold, salinity, drought, and heavy metal	Siji	Transcript-derived fragments viz., *TDF4*, *7*, *23*, *45*, *49*, *50*, *57*, *91* and *92*	Oligo-dT cDNAstart codon targeted marker (cDNA–SCoT) analysis and gene expression analysis using qRT-PCR	leaves and stems at various time points (0, 24, 48, and 72 h)	Fruit ripening, post-harvest storage, energy metabolism metabolite transport, post-transcriptional regulation of genes, flowering time control, plant defense, and abiotic stress responses	[60]

## Data Availability

Not applicable.
